# METABOLOMIC PROFILES FOR THE EXPLORATION OF BIOMARKERS IN SEVERE SARCOPENIA AMONG OLDER MEN

**DOI:** 10.1093/geroni/igac059.2321

**Published:** 2022-12-20

**Authors:** Hyung Eun Shin, Miji Kim, Daehyun Lee, Jae Young Jang, Chang Won Won

**Affiliations:** Kyung Hee University, Seoul, Seoul-t'ukpyolsi, Republic of Korea; Kyung Hee University, Seoul, Seoul-t'ukpyolsi, Republic of Korea; Kyung Hee Univerisity, Seoul, Seoul-t'ukpyolsi, Republic of Korea; Kyung Hee University, Seoul, Seoul-t'ukpyolsi, Republic of Korea; Kyung Hee University, Seoul, Seoul-t'ukpyolsi, Republic of Korea

## Abstract

The pathophysiology of sarcopenia is complex and multifactorial; however, it has not been fully elucidated. This preliminary study explored novel biomarkers of severe sarcopenia through a metabolomic analysis of plasma metabolites in community-dwelling older men. Twenty older men (mean age: 81.9±2.8 years) were randomly selected from the Korean Frailty and Aging Cohort Study. Participants with severe sarcopenia were compared to healthy, age-, and body mass index-matched controls (n = 10 each). Severe sarcopenia was diagnosed using the Asian Working Group for Sarcopenia 2019 criteria. Non-targeted metabolomic profiling of plasma metabolites was performed using capillary electrophoresis time-of-flight mass spectrometry. Among the 191 plasma metabolic peaks, 10 metabolites differed significantly between healthy controls and participants with severe sarcopenia. The plasma concentrations of l-alanine, homocitrulline, n-acetylserine, gluconic acid, n-acetylalanine, proline, and sulfotyrosine were higher, while the concentrations of 4-methyl-2-oxovaleric acid, 3-methyl-2-oxovaleric acid, and tryptophan were lower in participants with severe sarcopenia than in healthy controls (all, p < 0.05). Of the 57 metabolites quantified in target metabolites, L-alanine (area under the receiver operating characteristic curve [AUC] = 0.760, p = 0.049), gluconic acid (AUC = 0.800, p = 0.023), proline (AUC = 0.785, p = 0.031), and tryptophan (AUC = 0.800, p = 0.023) predicted the presence of severe sarcopenia. In conclusion, plasma metabolomic analysis demonstrated significant changes in amino acid, arginine, proline, and pentose phosphate metabolism in participants with severe sarcopenia. The identified metabolites could be helpful in understanding the underlying pathophysiology of sarcopenia.

